# The Role of Antimicrobial Susceptibility and Resistance Testing for *Mycoplasma genitalium* in Clinical Care

**DOI:** 10.1093/ofid/ofaf798

**Published:** 2026-03-30

**Authors:** Ken B Waites, Li Xiao, T Prescott Atkinson, William M Geisler

**Affiliations:** Department of Pathology, Heersink School of Medicine, University of Alabama at Birmingham, Birmingham, Alabama, USA; Department of Medicine, Heersink School of Medicine, University of Alabama at Birmingham, Birmingham, Alabama, USA; Department of Pediatrics, Heersink School of Medicine, University of Alabama at Birmingham, Birmingham, Alabama, USA; Department of Medicine, Heersink School of Medicine, University of Alabama at Birmingham, Birmingham, Alabama, USA

**Keywords:** antimicrobial resistance testing, fluoroquinolones, macrolides, *Mycoplasma genitalium*, sexually transmitted infections

## Abstract

*Mycoplasma genitalium* is a fastidious slow-growing bacterium that can be found in the lower urogenital tract of a small percentage of sexually active men and women in the general population. Its carriage in asymptomatic persons, particularly women, contributes to delayed diagnosis, spread of infection, and chronicity in some individuals. Thanks to improved detection methods, there is evidence that *M genitalium* is an important urogenital pathogen in causing inflammatory disease in men and women, which may have implications in infertility, pregnancy outcomes, and risk for HIV-1 acquisition. As more is learned about *M genitalium* and its pathogenic potential, laboratory testing to identify people with infections caused by this organism and selection of appropriate treatment regimens become increasingly important. However, these are challenging and complex matters due to the inherent difficulties in its detection in clinical specimens, rapidly escalating antimicrobial resistance, and limited classes of antimicrobial agents that are likely to be effective. The use of nucleic acid amplification tests that have the capability for detecting mutations conferring drug resistance in treatment decisions, referred to as resistance-guided treatment, is now recommended by public health organizations in multiple countries where such testing is available and has the potential to improve treatment outcomes.

## Teaser

*Mycoplasma genitalium* is a sexually transmitted urogenital pathogen that causes inflammatory disease and is challenging to treat. This review discusses laboratory methods for *M genitalium* detection, susceptibility and resistance testing, and the role of these methods in *M genitalium* management.

## *MYCOPLASMA GENITALIUM* AS AN AGENT OF HUMAN UROGENITAL DISEASE

*Mycoplasma genitalium* is a sexually transmitted pathogen that was first identified in the early 1980s in men with nongonococcal urethritis (NGU) [1]. Epidemiologic information about *M genitalium* infections is somewhat limited because, unlike other sexually transmitted infections (STIs) such as gonorrhea, chlamydia, and syphilis, reporting *M genitalium* infection to public health authorities is not required and there is no recommendation for screening asymptomatic individuals. *M genitalium* can be detected in the lower urogenital tract of about 1% to 7% of men and women in the general population but up to 38% in persons who manifest urogenital inflammatory symptoms and in those who attend sexual health clinics [2–6]. Coinfections with other sexually transmitted pathogens are common [7]. Occurrence of *M genitalium* in asymptomatic persons, especially women, contributes to delayed diagnosis, spread of infection, and chronicity in some individuals.

*M genitalium* causes 20% to 35% of all acute cases of NGU and up to 40% in men with recurrent or persisting NGU following antimicrobial treatment [5, 8]. Although *M genitalium* may be the cause of some cases of epididymitis, balanoposthitis, chronic prostatitis, and proctitis, its association with male infertility is not as convincing [5, 9, 10].

*M genitalium* may cause up to 30% of cases of cervicitis and endometritis and up to 33% of cases of pelvic inflammatory disease (PID), and it may be associated with bacterial vaginosis, dysuria, and tubal infertility [5, 8, 10–13]. Some investigations have found an association between *M genitalium* infections and ectopic pregnancy, preterm birth, stillbirth, and low birthweight, but findings are not consistent across all studies [11, 13–16].

*M genitalium* has been detected in the pharynx and rectum of men who have sex with men (MSM) and occasionally from these sites in women [17]. There are also a few reports of its detection outside of the urogenital tract in the joints of persons with arthritis and in the eyes of persons with conjunctivitis, but there have been no studies to determine the frequency with which such infections occur [18, 19].

Persistent infection of *M genitalium* in the urogenital tract is occasionally encountered in persons with a normal host immune system, but it may be especially problematic in persons who are immunosuppressed. We are following a young adult male who has X-linked agammaglobulinemia and a mild complement deficiency (mannose-binding lectin). He first developed NGU with dysuria and urethral discharge in November 2017 and was documented to have *M genitalium* by polymerase chain reaction (PCR). Despite attempts to treat the infection with tetracyclines, fluoroquinolones, and finally pristinamycin, his infection has remained stubbornly persistent into 2025, as documented by >30 positive PCR results over that 8-year period, showing very high levels of infection. The organism developed sequential resistance mutations during treatment to fluoroquinolones (it was originally macrolide resistant) and then to pristinamycin. Although he has become asymptomatic over time, his urinalysis continues to show evidence of significant inflammation, which is concerning for possible long-term consequences. This case provides evidence that humoral defects promote persistence of *M genitalium* infection, suggesting a mechanism for persistence in ostensibly healthy individuals. Even in the presence of a severe humoral defect, the organism did not produce an invasive extragenitourinary infection in this case.

*M genitalium* occurs more often at the urethral and rectal sites of MSM with HIV-1 than at those sites in MSM without HIV [20]. *M genitalium* cervicitis also occurs more often in persons with HIV than in women without HIV [21], and infection can persist longer in women with HIV [22]. *M genitalium* infections have additionally been associated with enhanced HIV transmission [5, 23].

A comprehensive description of the role of *M genitalium* as a human pathogen has been summarized in recent reviews [5, 10]. As more is learned about *M genitalium*, laboratory detection and selection of appropriate treatments become increasingly important. However, this is challenging and complex due to the inherent difficulties in its detection in clinical specimens, the rapid development and spread of acquired antimicrobial resistance, and the limited classes of antimicrobial agents that are likely to be effective. Public health organizations in multiple countries now recommend implementation of nucleic acid amplification tests (NAATs) that can detect mutations conferring drug resistance to guide antimicrobial selection as part of treatment decisions, referred to as resistance-guided therapy (RGT), which has the potential to improve treatment outcomes [6, 24–26].

## LABORATORY METHODS FOR *M GENITALIUM* DETECTION

The main reason why many years passed between the initial detection of *M genitalium* in men with NGU and the appearance of solid evidence implicating this organism in various inflammatory urogenital syndromes was the lack of reliable methods for its detection in clinical specimens. In the premolecular era, reliance on culture yielded dismal results. Early attempts at serology were hampered by cross-reactions with *Mycoplasma pneumoniae*. Antibody measurement for *M genitalium* has never been standardized or commercialized.

The fastidious nature and very slow growth of *M genitalium* necessitate several weeks to months for growth in culture to become evident. Previous authors described an enhanced culture technique using initial inoculation of specimens into Vero cells with subsequent monitoring for mycoplasmal growth by PCR, gradually adapting the organisms to grow in mycoplasmal culture media [27]. Isolation of *M genitalium* following Vero cell coculture was most likely to be successful in specimens with initial bacterial loads ≥10 000 genome equivalents [28]. Thus, culture can never be considered a practical means for patient diagnosis and management. Consequences of the lack of culture as a means for detection are the lack of clinical isolates for in vitro susceptibility testing, genomic characterization, and development of improved diagnostic assays [28].

NAATs were developed in the 1990s and utilized targets such as MgPa operon, 16S ribosomal RNA gene, *gap* encoding glyceraldehyde-3-phosphate dehydrogenase, and various other genes [5, 29]. There are numerous commercial NAATs sold outside the United States that not only detect *M genitalium*, often with various other sexually transmitted pathogens, but have the CE-IVD mark (Conformité Européenne marking on in vitro diagnostic medical devices); however, they have not been extensively evaluated in large, costly, and time-consuming clinical trials in comparison with one another or against reference methods, as would be required in the United States to be considered for Food and Drug Administration (FDA) clearance. A recent review described the evolution and current status of the laboratory detection of *M genitalium* with an emphasis on NAATs, which are now the methods of choice for detection [5].

There are 4 commercial NAATs for detection of *M genitalium* that have FDA clearance and are available for use in the United States [5]. The Aptima *Mycoplasma genitalium* (Hologic, Inc), the cobas TV/MG (Roche Molecular Systems), and the Alinity m STI (Abbott Molecular Diagnostic) are fully automated, sample-to-result assays that include nucleic acid extraction and provide qualitative results within a few hours. However, they are highly complex and expensive tests that cannot be readily performed in an ambulatory clinic setting. The Aptima *Mycoplasma genitalium* is an RNA-based NAAT that has been shown to provide excellent clinical sensitivity in organism detection when compared with various commercial and laboratory-developed DNA-based NAATs [5]. The cobas TV/MG has been evaluated in comparison with laboratory-developed PCRs and the Aptima *Mycoplasma genitalium* with comparable performance characteristics [30, 31]. The multiplex Alinity m STI performance has been shown to be comparable to laboratory-developed PCR assays and the S-DiaMGRes (Diagenode) [32]. The cobas Liat Ct/Ng/Mg Test System (Roche Molecular Systems) has a Clinical Laboratory Improvement Amendments–waived complexity classification and the capability for testing single samples with results available in about 20 minutes, making it the first true point-of-care (POC) test for *M genitalium* detection available in the United States. Recommended specimens for *M genitalium* detection are first-catch urine for males and vaginal swabs for females [5]. Other specimen types may be useful in some circumstances, such as rectal swabs, male penile or urethral meatal swabs, cervical swabs, prostatic biopsy tissues, epididymal fluid, and fallopian tube specimens [5].

## INDICATIONS FOR LABORATORY TESTING FOR *M GENITALIUM*—WHAT THE GUIDELINES SAY

A lack of available commercial NAATs for detection of *M genitalium* and general unfamiliarity among clinicians and public health organizations with this organism and its disease implications hindered *M genitalium* testing prior to 2015 [33]. In 2015, the US Centers for Disease Control and Prevention (CDC) added *M genitalium* as an emerging issue in its STI treatment guidelines, which discussed diagnostic considerations in the absence of an FDA-cleared NAAT [33]. The first FDA-cleared NAAT then became available for commercial use in 2019 [5]. In 2021, the CDC published new guidelines with recommendations for using *M genitalium* NAATs in specific clinical circumstances [26]; these guidelines were published several years after calls had begun for more widespread diagnostic testing, including resistance testing [29, 34].

The availability of commercial NAATs, some of which included resistance testing, in Europe and other regions led to more comprehensive diagnostic and management recommendations, including RGT, several years earlier than in the United States. Table 1 shows comparisons of diagnostic testing recommendations for the United States, United Kingdom, Europe, and Australia. US guidelines have not been emphatic regarding the need for testing at initial presentation of inflammatory urogenital conditions (urethritis, cervicitis, PID) caused by *M genitalium*. Yet, all 4 guidelines agree that symptomatic people who test positive for one of the inflammatory conditions associated with *M genitalium* and their sexual contacts should receive antimicrobial treatment due to the likelihood that failure to eradicate the organisms may lead to recurrent or persistent disease [25] and facilitate further spread of infections. UK and European guidelines specify that macrolide resistance testing be performed on all persons with positive NAAT findings, with consideration for fluoroquinolone resistance testing in cases of moxifloxacin treatment failure. US and Australian guidelines merely state that macrolide resistance testing be performed if available. Resistance testing on a broad scale is still unrealistic in the United States since there are no commercial FDA-cleared macrolide resistance NAATs in early 2026 and such testing is limited to a few reference laboratories using their own internally validated procedures. Owing to concern for overtreatment, costs to the health care system, further selection and spread of antimicrobial resistance, and a lack of knowledge about long-term outcomes of asymptomatic carriage, there are no guidelines that recommend testing asymptomatic people for *M genitalium* [13].

**Table 1.** Comparison of Current Recommendations From 4 Published Guidelines for Performance of Diagnostic Tests for *Mycoplasma genitalium*

NAAT Testing Recommended	United States 2021 [26]	United Kingdom 2025 [6]	Europe 2021 [25]	Australia 2023 [24]
Asymptomatic persons	No	No	Not specified	No
Test of cure following treatment of people who test positive	Only if resistance testing is not available and moxifloxacin cannot be used	Only if a person remains symptomatic after treatment when there is suspicion of persistent infection	Consider, no earlier than 3 wk	No earlier than 14–21 d after completion of treatment and only if symptoms persist and/or there is ongoing risk of reinfection or sequelae
Sex partners of symptomatic people who test positive	Yes	Yes	Yes	Yes
Resistance testing on all people who test positive	Yes, if available^a^	Yes (macrolide)^b^	Yes (macrolide)^b^	Yes, if available (macrolide)
**Men**				
Nongonococcal urethritis	Yes, if persistent or recurrent after treatment	Yes	Yes	Yes
Epidididymo-orchitis	Not specified	Yes^c^	Yes, if age <50 y	Not specified
Sexually acquired proctitis	Consider if symptoms persist after treatment	Yes^c^	Yes^d^	Yes
**Women**				
Mucopurulent cervicitis	Yes, if recurrent	Yes^c^	Yes	Yes
Symptoms of pelvic inflammatory disease	Consider	Yes	Yes	Yes
Intermenstrual or postcoital bleeding	Not specified	Yes^c^	Yes	Yes
Dysuria with no known other etiology	Not specified	Not specified	Yes	Not specified
Sexually acquired proctitis	Consider if symptoms persist after treatment	Yes^c^	Yes^d^	Yes
Termination of pregnancy	Not specified	Not specified	Consider	Not specified

Abbreviation: NAAT, nucleic acid amplification test.

^a^In 2026 there were no Food and Drug Administration–cleared NAATs commercially available in the United States that incorporated resistance testing. Resistance testing for macrolides and fluoroquinolones is available through the UAB Diagnostic Mycoplasma Laboratory in Birmingham, Alabama, which uses validated laboratory-developed polymerase chain reaction assays.

^b^Test for fluoroquinolone resistance in cases of moxifloxacin treatment failure.

^c^If symptoms persist, other infections have been ruled out, and index of suspicion is high.

^d^If *Chlamydia trachomatis* and *Neisseria gonorrhoeae* are excluded.

## ANTIMICROBIAL SUSCEPTIBILITY PROFILES

Table 2 provides susceptibility profiles for various antimicrobial agents grouped according to their classes that have been tested in vitro against *M genitalium*. Individual listings include only those drugs that have been licensed or are currently available for clinical use in some countries. Methods by which minimal inhibitory concentrations (MICs) were determined have not been standardized and may differ from one report to another. The first reports of in vitro susceptibilities were based on MIC testing with broth microdilution or agar dilution testing of very small numbers of reference strains and clinical isolates and were performed prior to the emergence of antimicrobial resistance. More recent MIC data were often obtained from the Vero cell coculture system with quantitative PCR to monitor bacterial growth in the presence of antimicrobial agents [35, 36]. Epidemiologic reports describing resistance rates for macrolides and fluoroquinolones are based primarily on genotypic testing of clinical specimens for *M genitalium* gene mutations known to be associated with elevated MICs or treatment failures rather than direct measurements of MICs.

**Table 2.** MIC Ranges for Various Antimicrobials Tested In Vitro Against *Mycoplasma genitalium*

Antimicrobial	MIC Range, µg/mL	Selected References
**Tetracycline** ^a^		
Tetracycline	≤0.01–32	[35–43]
Doxycycline	0.004–2.5	[35–37, 39, 40, 42, 44–51]
Eravacycline	0.002–2	[38]
Minocycline	≤0.01–2.5	[36, 37, 42, 51]
Omadacycline	0.063–0.5	[37]
**Macrolide**		
Erythromycin^b^	≤0.01 to >64	[35, 42, 44, 52]
Clarithromycin^b^	≤0.001–128	[35, 36, 39, 42, 43]
Azithromycin^b^	≤0.001–250	[35–37, 39–45, 48, 49, 51, 52]
Josamycin^c^	0.02	[40]
**Lincosamide**		
Clindamycin	0.2–5	[40, 42, 43]
Lincomycin	1–25	[40, 42]
**Pleuromutilin**		
Lefamulin^b,d^	≤0.001–0.064	[51–53]
**Streptogramin**		
Pristinamycin^d,e^	<0.01–0.02	[40]
**Amphenicol** ^f^		
Chloramphenicol	0.5–25	[40, 42, 54]
Thiamphenicol	2–8	[55]
**Aminocyclitol**		
Spectinomycin^g^	1–5	[42, 55]
**Fluoroquinolone** ^d^		
Ciprofloxacin	0.063 to >16	[35, 36, 39, 40, 42–44]
Levofloxacin	0.5–8	[35–37, 39, 41, 49–51]
Moxifloxacin	0.03 to >16	[35–39, 41, 44, 46–48, 52]
Sitafloxacin^h^	0.008–0.25	[36, 56]
**Triazaacenaphtylene**		
Gepotidacin^b,d^	≤0.016–0.5	[41, 48]
**Spiropyrimidinetrione**		
Zoliflodacin^i^	0.125–4	[49, 57]
**Nitroimidazole**		
Metronidazole	6.3–12.5	[58]
Secnidazole	3.1–12.5	[58]
Tinidazole	0.8–6.3	[58]
**Fusidane**		
Fusidic acid	0.63–2.5	[46]

Abbreviation: MIC, minimal inhibitory concentration.

^a^Includes tetracycline-resistant isolates with MICs ≥8 µg/mL.

^b^Includes azithromycin-resistant isolates with MICs ≥2 µg/mL.

^c^Includes only macrolide-susceptible isolates.

^d^Includes moxifloxacin-resistant isolates with MICs ≥2 µg/mL.

^e^Pristinamycin is available in some European countries and by special arrangement in Australia.

^f^Chloramphenicol and thiamphenicol are not available in the United States but may have availability in other countries.

^g^Spectimomycin is still licensed in a few countries outside the United States but is unlikely to be available as of 2026.

^h^Sitafloxacin is available in Japan and by special arrangement in Australia.

^i^Zoliflodacin was approved in the United States in Dccember 2025 for treatment of uncomplicated gonorrhea.

As there are no designated MIC breakpoints for *M genitalium*, in vitro susceptibility data are merely reported as MIC values. Resistance is deemed present as follows: when phenotypic MICs are multiple dilutions higher than those of wild type (WT) reference strains (usually >1 µg/mL); when documented by the presence of gene mutations that have been associated with acquired resistance in other bacterial species; and in cases of poor microbiological and clinical efficacy when antimicrobials are used to treat *M genitalium* infections.

Due to the lack of a cell wall, antimicrobial classes with potential activity against *M genitalium* have been limited mainly to those agents that exert their effects by inhibition of either protein or nucleic acid synthesis. Many agents shown to have potentially acceptable in vitro activity (MICs ≤1 µg/mL) have never been used to treat *M genitalium* infections in clinical practice. An example is the ketolide solithromycin, which has high in vitro potency against *M genitalium*, including strains demonstrating resistance to macrolides [44]. However, due to safety concerns and side effect profiles, solithromycin has not been approved for clinical use. Clindamycin is active in vitro, but there is no clinical evidence that it works in vivo.

Drug classes available in the United States for *M genitalium* treatment that have achieved widespread utilization are tetracyclines, macrolides, and fluoroquinolones. Even though the first *M genitalium* isolates appeared to be susceptible to agents in all 3 classes, clinical response to doxycycline has always been poor, about 30%–40% clinical cure, and microbiological response may be even lower [45], despite the lack of evidence of elevated MICs.

In vitro elevation of MICs to tetracycline has recently been documented in a few *M genitalium* isolates (MICs, 8–32 µg/mL). Tetracycline resistance in *M genitalium* is not mediated by *tet(M)* as it is in *Mycoplasma hominis* and *Ureaplasma* species and appears to be due to active efflux in some instances [37, 38]. Single-nucleotide polymorphisms (SNPs) in *M genitalium* 16S rRNA that have been shown to be associated with tetracycline resistance in other bacteria have been detected, but no clear relationships have been identified thus far between their presence and doxycycline MICs or organism load in persons receiving doxycycline treatment [59–61].

MICs for a new minocycline derivative, omadacycline, were low and unaffected by tetracycline resistance (MICs, 0.063–0.125 µg/mL) [37]. Although omadacycline is FDA approved for the treatment of community-acquired bacterial pneumonia and acute skin and skin structure infections, there are no clinical data to determine how well it performs in vivo against *M genitalium*. Eravacycline is a synthetic fluorocycline approved to treat complicated abdominal infections, and it shows greater in vitro potency against *M genitalium* than tetracycline [38]. A lack of oral formulation limits its attractiveness for treating STIs.

Minocycline has a good tolerability profile. It has similar MICs to doxycycline [36] and can be effective in treating macrolide-resistant *M genitalium* infections that have not responded to other agents [62]. One study reported a 67% microbiological cure rate for macrolide-resistant infections when minocycline was used as monotherapy [63]. When used sequentially after doxycycline, the cure rate was 90.4% [64].

In view of relatively poor efficacy of doxycycline, emphasis had been placed on macrolides and fluoroquinolones as first- and second-line treatments, respectively. However, over the past 15 years, the emergence of high rates of macrolide resistance has led to azithromycin no longer being a first-line option. This fueled the idea for the RGT approach with moxifloxacin and doxycycline. Doxycycline is typically used in sequential combination with azithromycin or moxifloxacin to enhance organism eradication by lowering the initial bacterial load and lessening the chance for resistance selection [13, 26].

SNPs that result in elevation of macrolide MICs (MRMs) are concentrated in region V of the 23S rRNA gene. The 5 SNPs that are known to confer macrolide resistance are A2058G, A2058C, A2058T, A2059G, and A2059C (*Escherichia coli* nucleotide numbering) [5, 65]. There is a strong correlation between MRMs and elevated azithromycin MICs as well as clinical and microbiological treatment failures [26, 45].

SNPs in the quinolone resistance–determining region of the topoisomerase IV subunit gene *parC*, such as G248T, result in amino acid changes in ParC protein (S83I, *M genitalium* amino acid numbering), with a corresponding increase in the MIC and a moxifloxacin treatment failure rate of about 60% [5, 65–69]. Other SNPs in *parC*, which occur less often and are not as closely associated with treatment failure, include G248A (S83N) and G259A/T/C (D87N, D87Y, D87H, respectively) [70]. Amino acid changes in the quinolone resistance–determining region of DNA gyrase subunit GyrA include G285A/T (M95I) and G295T/A (D99Y/N) and may double the rate of moxifloxacin failures when combined with a ParC S83I change [68, 69]. Mutations in *gyrA* rarely occur without concomitant mutations in *parC*. In the absence of quinolone resistance mutations (QRMs), moxifloxacin is nearly 100% effective in curing *M genitalium* infection [71]. Very little is known about the potential contributions of mutations in *parE* or *gyrB* to moxifloxacin resistance in *M genitalium*, and these targets have not been studied to a significant degree.

The prevalence and type of MRMs and QRMs vary geographically, as well as by population surveyed, STI history, prior antimicrobial use, regional and national antimicrobial prescribing practices, and sexual preferences. MRMs occur more often in people presenting to sexual health clinics, especially MSM, likely reflective of frequent treatments with macrolides for various STIs [61, 72–77]. Reported resistance rates can also vary depending on the methods of detection: laboratory-developed tests, commercial NAAT kit, Sanger sequencing, and the MRMs and QRMs evaluated.

A recent global review [65] reported macrolide, fluoroquinolone, and dual-class resistance rates of 33%, 13.3%, and 6.5%, respectively. Macrolide resistance was highest in Europe (56.8%), followed by the Americas (53%), Western Pacific (46.5%), and Africa (0.13%). Individual country prevalence for MRMs ranged from 0% to 86.7%. The prevalence of QRMs was highest in the Western Pacific (40.5%), followed by Europe (12.3%), the Americas (5.5%), and Africa (5.1%). Country-specific QRM prevalence ranged from 0% to 73.3%. Dual-class resistance was highest in the Western Pacific (29%), followed by Europe (7.3%), the Americas (4.3%), and Africa (0%).

Some older antimicrobial agents are potential third-line alternatives (Table 3) [6, 24, 25]. These agents, for which in vitro activities are shown in Table 2, include minocycline (discussed previously), josamycin, pristinamycin, and sitafloxacin.

**Table 3.** Comparison of Current Treatment Recommendations for *Mycoplasma genitalium* From 4 Published Guidelines^a^

Treatment Regimen	United States 2021 [26]	United Kingdom 2025 [6]	Europe 2021 [25]	Australia 2023 [24]
**Uncomplicated infection**				
First line				
MRM not determined	Dox 100 mg 2×/d × 7 d, followed by Mox 400 mg 1×/d × 7 d ^b^	Dox 100 mg 2×/d × 7 d, followed by Azi 1 g × 1 d, then Azi 500 mg 1×/d × 2 d	Azi 500 mg × 1 d, followed by Azi 250 mg × 4 d or josamycin 500 mg 3×/d × 10 d	Dox 100 mg 2×/d × 7 d, followed by Azi 1 g × 1 d, then Azi 500 mg 1×/d × 3 d
MRM not detected	Dox 100 mg 2×/d × 7 d, followed by Azi 1 g × 1 d, then Azi 500 mg 1×/d × 3 d	Dox 100 mg 2×/d × 7 d, followed by Azi 1 g × 1 d, then Azi 500 mg 1×/d × 2 d	Azi 500 mg × 1 d, followed by Azi 250 mg × 4 d or josamycin 500 mg 3×/d × 10 d	Dox 100 mg 2×/d × 7 d, followed by Azi 1 g × 1 d, then Azi 500 mg 1×/d × 3 d
Second line				
MRM detected or Azi treatment failure	Dox 100 mg 2×/d × 7 d, followed by Mox 400 mg 1×/d × 7 d	Dox 100 mg 2×/d × 7 d, followed by Mox 400 mg 1×/d × 7 d	Mox 400 mg 1×/d × 7 d	Dox 100 mg 2×/d × 7 d, followed by Mox 400 mg 1×/d × 7 d
Third line				
After second-line treatment failure or drug intolerance	Not specified	Dox 100 mg 2×/d × 7 d, followed by Pri 1 g 3×/d × 10 d or Min 100 mg 2×/d × 14 d	Min 100 mg 2×/d × 14 d or Dox 100 mg 2×/d × 14 d or Pri 1 g 4×/d × 10 d	Pri 1 g 3×/d with Dox 100 mg 2×/d × 10 d or Min 100 mg 2×/d × 14 d or sitafloxacin 100 mg 2×/d with Dox 100 mg 2×/d × 7 d
**Complicated infection**				
PID	Mox 400 mg 1×/d × 14 d	Mox 400 mg 1×/d × 14 d or Azi 1 g × 1 d, followed by Azi 500 mg 1×/d × 4 d	Mox 400 mg 1×/d × 14 d	Mox 400 mg 1×/d × 14 d
Epididymo-orchitis	Not specified	Same as for PID	Same as for PID	Not specified

Abbreviations: Azi, azithromycin; Dox, doxycycline; Min, minocycline; Mox, moxifloxacin; MRM, macrolide resistance mutation; NAAT, nucleic acid amplification test; PID, pelvic inflammatory disease; Pri, pristinamycin.

^a^Treatment regimens for symptomatic persons who have tested positive for *M genitalium* by NAAT and their sexual contacts.

^b^If Mox cannot be used as a first-line treatment regimen for patients for whom MRM testing is not available, an alternative regimen to consider is Dox (100 mg 2×/d × 7 days), followed by Azi (1 g × 1 day; then, 500 mg 1×/d × 3 days), with a test of cure 21 days after completion of therapy.

Josamycin is a 16-membered macrolide available in some European and Asian countries but not the United States. It has low MICs against macrolide-susceptible *M genitalium* (0.02 µg/mL) and is a treatment alternative in the European guidelines when MRMs are not present [25]. Josamycin was used for treating macrolide-susceptible *M genitalium* in Russian men with NGU, with a 93.5% eradication rate, but MRMs were rapidly selected during treatment [78].

Pristinamycin is a mixture of a macrolide and a streptogramin that produces a synergistic antibacterial effect. It is available in some European countries and in Australia by special arrangement but not in the United States. Pristinamycin has been used as monotherapy or in combination with doxycycline to treat macrolide-resistant *M genitalium* infections, yielding a cure rate of 75%, making it a reasonable alternative [79]. A pristinamycin treatment failure was likely caused by a 23S rRNA mutation at position 2062 [80].

Sitafloxacin is a fourth-generation fluoroquinolone available in Japan and Australia but not in Europe or the United States. It is included as a third-line drug used in combination with doxycycline in the Australian treatment guidelines [24]. Sitafloxacin has low MICs against *M genitalium* (0.008–0.25 µg/mL), even strains with QRMs, and has been used as monotherapy and in combination with doxycycline or minocycline in persons with QRMs and prior moxifloxacin treatment failures [64, 68, 81–84]. When used as monotherapy, sitafloxacin resulted in microbiological cure in 92.2% of strains with the G248T (S83I) mutation and WT *gyrA* but in only 41.7% of those containing *parC* and *gyrA* mutations, suggesting that this drug may be of particular value in the absence of *gyrA* mutations [85]. A cure rate of 68.8% for *M genitalium* harboring *parC* and *gyrA* mutations was achieved with a longer 21-day course of sitafloxacin combined with doxycycline [81]. Whether treatment failures have resulted in higher MICs because of antimicrobial exposure has not been determined.

There are a few other older antimicrobials that demonstrate potentially acceptable in vitro potencies that are now receiving renewed consideration. MIC data for amphenicols are sparse and their use has been extremely limited. A UK man with persistent NGU whose doxycycline and moxifloxacin treatments failed was given chloramphenicol for 14 days and achieved microbiological cure with symptom resolution [86]. Concern for blood dyscrasias led to revocation of chloramphenicol approval for oral use in the United States, although it may still be obtained in some other countries because of its minimal expense. Thiamphenicol, which may be less toxic than chloramphenicol, has been considered a possible option as it has been used successfully to treat urethritis and PID [87, 88]. Recent in vitro data suggest that thiamphenicol may be useful in the treatment of some strains of MDR *M genitalium* and may exhibit synergism with doxycycline [89]. Thiamphenicol has very limited availability in a few countries, not including the United States [55].

The aminocyclitol spectinomycin has been used to treat gonorrhea. MIC data are sparse [55] but suggest that it may be an alternative for MDR *M genitalium*. A case of macrolide-resistant *M genitalium* NGU was successfully treated with spectinomycin [90]. Spectinomycin is not available in the United States. Although it is still registered in some European countries, it is no longer distributed and may be difficult or impossible to obtain. Requirement for daily painful intramuscular injections for at least a week further limits its utility for STIs.

Recent in vitro data for various nitroimidazoles and fusidic acid are encouraging as potential new therapeutic options for MDR infections [46, 58]. Tinidazole has shown promise in combination with minocycline for treatment of macrolide-resistant *M genitalium* infections (80% microbiological cure) [58, 91]. Metronidazole in combination with doxycycline was more effective in eradicating *M genitalium* in women with PID than doxycycline monotherapy [92].

There was a recent report of 5 men with NGU caused by *M genitalium* whose treatment with multiple other agents had failed. They were treated with a quadruple combination of methenamine, pristinamycin, metronidazole, and minocycline for 14 to 28 days and all achieved microbiological cure. This concept of using multiple combinations of antimicrobials has become the cornerstone of other hard-to-treat infections caused by pathogens such as *Helicobacter pylori* and *Mycobacteria* spp and may deserve further considerations for *M genitalium* [93].

Given the limited efficacy and availability of existing antimicrobials, the need for new drugs with different targets not affected by cross-resistance is great. Three agents have been evaluated in vitro, 1 of which has undergone limited clinical evaluation against *M genitalium*. Lefamulin is a pleuromutilin that is FDA approved for the treatment of community-acquired bacterial pneumonia. Lefamulin inhibits bacterial growth by binding at the 50S ribosome at a site distinct from macrolides. It is active in vitro against *M genitalium*, including strains with MRMs and QRMs (MICs ≤0.001–0.064 µg/mL) [47, 52, 53, 94]. A small number of patients who had previously experienced microbiological failure or could not be given moxifloxacin were randomized to receive lefamulin as monotherapy or with concurrent or sequential doxycycline. Outcomes were disappointing. Monotherapy for 7 days led to only 3 of 10 (30%) microbiological cures while the 14-day course yielded 2 of 3 (67%) cures. Sequential or concomitant treatment with doxycycline resulted in microbiological cure in only 6 of 14 (43%) cases. Gastrointestinal side effects occurred in most patients [47]. Lefamulin is not currently available anywhere and its future is uncertain.

Gepotidacin is a new triazaacenaphtylene that is FDA cleared for the treatment of urinary tract infections. It inhibits DNA replication by inhibition of topoisomerase II with a target and mechanism distinct from those of fluoroquinolones. It has good in vitro activity against *M genitalium* with MRMs and/or QRMs (MICs ≤0.5 µg/mL). Gepotidacin was also bactericidal and synergistic with doxycycline in a small number of isolates [48]. There are no data to demonstrate clinical efficacy against *M genitalium.*

Zoliflodacin is a spiropyrimidinetrione topoisomerase II inhibitor approved for treament of uncomplicated treat gonorrhea, which has been evaluated in vitro against *M genitalium* [49, 57]. Zoliflodacin was active against most strains (MIC_90_ = 0.5 µg/mL), including those with MRMs and QRMs. Only 1 of 47 (2.1%) strains had an elevated zoliflodacin MIC (4 µg/mL). There are no published data to demonstrate clinical efficacy against *M genitalium.*

## RESISTANCE TESTING METHODS

### Phenotypic MIC Measurement

The extremely slow growth of *M genitalium* makes it impossible to perform traditional MIC testing in accordance with guidelines published by the Clinical Laboratory Standards Institute [95]. However, once a strain has been grown and passaged sufficiently to become laboratory adapted and can be grown in SP4 broth to suitable concentrations, MIC measurement can be undertaken by broth microdilution with some adaptations to the procedure of the Clinical Laboratory Standards Institute. Details of this procedure used at the UAB Diagnostic Mycoplasma Laboratory for measuring in vitro antimicrobial activities against *M genitalium* are provided elsewhere [5 ].

MIC testing for *M genitalium* is performed primarily for evaluation of new antimicrobial agents. However, MIC measurement with clinical isolates has been proven useful in our laboratory for some cases of MDR *M genitalium* infections where there was treatment failure to search for potential alternative regimens.

The Vero cell/PCR culture technique [27] has also been used to determine MICs [35, 36, 48]. Growth of *M genitalium* in the presence of antimicrobials is determined by quantitative PCR rather than color change in broth as with traditional MIC measurements. The Vero cell/PCR assay is not internationally validated or approved for diagnostic use and may produce MICs that are higher for some antimicrobials when compared with the broth microdilution assay [35, 36].

### Genotypic Resistance Testing

Testing clinical specimens positive for *M genitalium* directly or reflexively for resistance mutations can provide information useful for RGT. Although time-consuming and laborious, sequencing of PCR products can potentially identify the presence of all mutations in the target regions. Sanger sequencing of the genes of interest is considered the reference method. Targeted next-generation sequencing is relatively high throughput and may be a direction for detecting resistance-associated mutations in *M genitalium* [96]. Other techniques can be used, such as pyrosequencing and real-time PCR coupled with high-resolution melting analysis, different primers targeting WT and mutants, labeled hydrolysis probes, and fluorescence resonance energy transfer probes coupled with melting curve analysis [7, 96–104]. Detection of *M genitalium* in clinical specimens and simultaneously testing for antimicrobial resistance mutations with one of these technologies is the most logical and desired approach from cost- and time-saving perspectives.

The UAB Diagnostic Mycoplasma Laboratory developed and validated a real-time PCR assay (MGMR) that simultaneously detects *M genitalium* and the 5 MRMs known to be associated with elevated azithromycin MICs based on a modification of the PCR described by Touati et al [98]. Details have been described elsewhere [105]. A second reverse transcriptase–quantitative PCR assay for detecting QRMs in the *parC* and *gyrA* genes was also developed and internally validated by the UAB laboratory: it covers all currently reported QRMs (12 *parC* mutations and 10 *gyrA* mutations) in both genes but avoids the most frequent synonymous mutation C234T in *parC*. When multiplexed with MGMR and QRMs, the combined assay enables simultaneous detection of *M genitalium* and identification of the clinically relevant MRMs and QRMS and can help to optimize treatment strategies in a more efficient fashion. It is available for clinical diagnostic testing in accordance with CDC treatment guidelines [26]. Drawbacks of this method are that it may report some rare mutations not associated with fluoroquinolone resistance, it is quite expensive and labor intensive, and it is a highly complex procedure not available elsewhere.

Commercial NAATs that incorporate organism and antimicrobial resistance mutation detection have been CE-IVD marked and sold in various countries (Table 4) but not the United States. Most detect only MRMs, but some kits that detect QRMs have also been developed. Although there are no NAATs that include resistance testing that are FDA cleared for use in the United States, some products can be purchased for detection of MRMs for research use only and internally validated so that they can be used to guide patient management.

**Table 4.** Commercial Assays Detecting Antimicrobial Resistance in *Mycoplasma genitalium* Available Outside the United States^a^

			Resistance Mutation Detection		
Product (Manufacturer)	Platform and Method	*M genitalium* Gene Targets	Macrolide	Fluoroquinolone	Selected References
ResistancePlus MG (SpeeDx)	Platform varies, PlexZyme, PlexPrime, PCR	*mgPa*	5 mutations in 23S rRNA: A2058G/C/T, A2059C/G	Not included	[76, 101, 106–116]
ResistancePlus MG FleXible (SpeeDx/Cepheid)	Cepheid GeneXpert, PlexZyme, PlexPrime, PCR	*mgPa*	4 mutations in 23S rRNA: A2058G/C/T, A2059G	Not included	[117–121]
Macrolide-R/MG ELITe MGB (ELITech Group)	ELITe InGenius, PCR	23S rRNA	5 mutations in 2S rRNA: A2058G/C/T, A2059C/G	Not included	[121]
Allplex MG & AziR (Seegene)	Bio-Rad Cfx96, PCR	Undisclosed	6 mutations in 23S rRNA: A2058G/C/T, A2059G/C/T	Not included	[108, 121]
Allplex MG & MoxiR Assay (Seegene)	Bio-Rad Cfx96, PCR	Undisclosed	Not included	6 mutations in *parC* (A247C, G248A/T, G259C/T/A)	[122]
AmpliSens *M genitalium-*ML/FQ-Resist-FL (Central Research Institute for Epidemiology)	Rotor-Gene, Qiagen, GmbH, PCR	*gyrB,* wild type 23S rRNA; wild type *parC*	None^b^	None^b^	[110, 123]

Abbreviation: PCR, polymerase chain reaction.

^a^Assays include only those that have received the CE-IVD mark (Conformité Européenne marking on in vitro diagnostic medical devices).

^b^This assay is based on the principle that amplification of wild type 23S rRNA and/or wild type *parC* genes determines the absence of mutations in these sites conferring resistance; thus, no actual mutations are detected.

Commercial kits produce results within 2 to 4 hours, so they cannot be considered POC tests. Their performances to detect *M genitalium* and resistance mutations have been evaluated by comparison with other commercial assays, laboratory-developed tests, and reference methods such as Sanger sequencing. A complete understanding of the capabilities of commercial NAATs that detect resistance is limited by the very small numbers of specimens in many of the published comparisons. An in-depth discussion of NAATs used for resistance detection is available in a recent review [5].

ResistancePlus MG has been the most extensively studied NAAT for MRM detection. Its sensitivity for *M genitalium* detection is somewhat lower than that of other assays that do not include resistance testing but is comparable to other kits that include resistance testing [106–108]. Sensitivity and specificity for MRM detection are similar to other kits and to reference methods [5, 101, 106, 109, 110]. ResistancePlus MG provides a result indicating whether resistant or WT genotype is present, while some products such as Allplex MG & AziR report the actual SNPs, which can be useful for surveillance purposes [108]. ResistancePlus MG FleXible has been adapted for use on the Cepheid GeneXPert, incorporating DNA extraction and amplification, real-time PCR detection of *M genitalium* and MRMs, and reporting of results in random access so that single specimens can be analyzed. ResistancePlus MG FleXible performed acceptably when compared with reference methods and other commercial kits [117–121]. A comparison was conducted among the ResistancePlus MG FleXible, Macrolide-R/MG ELITe MGB, and Allplex MG & AziR against a laboratory-developed PCR test and 23S rRNA gene sequencing as reference and found similar specificities for the 3 kits for MRM detection, but the Allplex MG & AziR kit missed detecting MRMs in 14.4% of *M genitalium*–positive specimens, a major concern for its reliability, warranting further evaluation [121].

Only 2 commercial NAATs for detection of QRMs have received the CE-IVD mark: the Allplex MG & MoxiR and the AmpliSens *M genitalium*-ML/FQ-Resist-FL (Table 4). The Allplex MG & MoxiR assay, which detects 6 *parC* mutations, was evaluated in comparison with 2 research use–only kits against specimens known to be positive for *M genitalium*, with Sanger sequencing as reference [122]. The Allplex MG & MoxiR clinical sensitivity and specificity for detection of QRMs were 91.8% and 100%, respectively.

The AmpliSens *M genitalium-*ML/FQ-Resist-FL is the first NAAT that includes reagents to detect macrolide and fluoroquinolone resistance [110]. This product includes 1 PCR that detects *M genitalium* by using the *gyrB* gene target and WT 23S rRNA gene. Another PCR detects an internal control and WT *parC.* Nonamplification of WT 23S rRNA and WT *parC* indicates the presence of MRMs and QRMs, although no actual mutations are detected. In one evaluation [123], there was 100% sensitivity and 99.2% specificity for the detection of potential MRMs and 92.3% sensitivity and 100% specificity for the detection of potential QRMs. A second study [110] evaluated this PCR against the S-DiaMGRes and ResistancePlus MG in a group of specimens with previously determined *M genitalium* results, with Sanger sequencing as reference. Sensitivity for *M genitalium* detection was comparable for all 3 assays (98.1%–100%). Sensitivities for detection of MRMs ranged from 90.7% to 100% while specificities were 88.6% to 100% for all 3 kits. Sensitivity and specificity were 100% for detection of QRMs.

The AmpliSens kit requires ≥1000 genomic equivalents in the specimen for a valid test and some clinical specimens have lower numbers than this. Lack of amplification of WT 23S rRNA and *parC* may also occur if mutations are present that have not been associated with elevated MICs or treatment failures, meaning that resistance may be overestimated. Neither assay incorporates reagents for detection of *gyrA* mutations such that opportunities will be missed to identify some people who are most likely to fail moxifloxacin treatment [68, 69].

## IS RESISTANCE-GUIDED TREATMENT WORTH THE EFFORT AND THE COST?

Use of azithromycin without resistance testing may result in treatment failures due to the high prevalence of MRMs. Use of moxifloxacin without documenting the presence of MRMs (to decide if azithromycin is a treatment option) is not ideal: moxifloxacin is more costly than azithromycin and has greater safety concerns, and its indiscriminate use can potentially limit future availability due to resistance development. In the absence of MRMs, azithromycin remains highly effective, just as moxifloxacin remains effective in the absence of QRMs. Optimizing outcomes and minimizing spread of drug-resistant infections through good antimicrobial stewardship with individualized treatment based on direct or reflexive resistance testing is desirable from patient care and epidemiologic standpoints and should be economically beneficial to the health care system. Lack of FDA-cleared NAATs to detect resistance means that such testing is rarely performed in the United States, even as RGT is becoming more widely utilized in Australia and Europe.

Prospective studies support the use of RGT to provide microbiological cure of infections, although the number of studies and patients enrolled in some of them are quite small. Read et al [124] and Durukan et al [125] described cohorts of Australian men and women who have tested positive for *M genitalium* and their sexual contacts, who were treated sequentially with doxycycline followed by azithromycin or with doxycycline followed by sitafloxacin or moxifloxacin. Microbiological cures occurred in >90%, representing an improvement over historical treatment success rates pre-RGT when azithromycin was used as monotherapy. Selection of macrolide resistance occurred in only 4.6%. A third study [67] also found beneficial results of RGT, with a cure rate of 93% for infections without MRMs treated with doxycycline/azithromycin and 85% for infections positive for MRMs treated with doxycycline/moxifloxacin. A small UK study [126] involved men and women who have tested positive for *M genitalium* and their partners, who were treated with sequential doxycycline/azithromycin or doxycycline/moxifloxacin depending on the presence or absence of MRMs. Treatment failure decreased overall with RGT to 3% from 27% pre-RGT (*P* = .008). However, improved therapeutic success with RGT was noted only in men with NGU and not for women with PID. A small study from Bulgaria [127] evaluated RGT to direct monotherapy with either doxycycline or moxifloxacin. MRMs were detected in 9 (50%) patients. Treatment failed in 1 person (5.9%) with macrolide-resistant *M genitalium* who was subsequently cured with pristinamycin. Although numbers are quite small, the 5.9% treatment failure rate with RGT was significantly less than the 47.6% failure rate pre-RGT (*P* = .002). Reduction of symptoms has not been a primary focus of studies evaluating the effect of RGT on microbiological outcomes, as they typically included asymptomatic sexual partners of index cases, symptoms may have resolved prior to organism clearance, and coinfections often occurred.

There are no recommendations to test specimens for QRMs except in cases of moxifloxacin failure (Table 1). Possible reasons include the relatively low frequency of moxifloxacin resistance in some regions [65]. NAATs capable of detecting QRMs have limited or no availability, and their performances are not well established. As there are several SNPs of potential clinical significance, sometimes with an inconsistent relationship with treatment failures, uncertainty remains regarding how to interpret their presence in the absence of corresponding MIC data [128]. There are no other recommended treatment options in some countries if moxifloxacin fails. Thus, knowing whether QRMs are present may not always affect treatment decisions. A final concern is cost for QRM testing in addition to MRM assays, which can be considerable because of the complexity, expenses for reagents and labor, and number of SNPs that must be evaluated [129].

Despite the favorable results described here for RGT based on detection of MRMs, moxifloxacin treatment failures caused by MRM-positive *M genitalium*, presumably related to the presence of undetected QRMs, can be quite high [130]. As prevalence of QRMs increases, reasons for moxifloxacin treatment failures are being critically examined. NAATs capable of detecting QRMs are expected to become more widely available. Hence, the need for including QRM testing as part of a “next-generation RGT algorithm” is now being considered in Australia [67, 68, 131, 132]. The ParC G248T (S83I) mutation was detected in 55.4% of *M genitalium*–positive specimens. Among them, more than half (56.9%) contained a concurrent mutation in GyrA [133]. This study has implications supporting the need for resistance testing that includes detection of ParC and GyrA changes because of potential synergism making treatment failures more likely when mutations in ParC and GyrA are present in the same specimen [68]. Moreover, the absence of the S83/D87 changes in *parC* is predictive of moxifloxacin cure in 96% to 98% of patients, indicative of the value of an assay to detect the WT sequence of *parC* to facilitate RGT [67, 132]. Use of RGT that includes detection of QRMs would be most practical in countries such as Australia, where there is a high rate of moxifloxacin resistance and third-line antimicrobials such as minocycline, pristinamycin, and sitafloxacin can be readily acquired.

RGT may improve microbiological outcomes with anticipated cure rates ≥96% for infections not associated with QRMs [131]. Targeted use of azithromycin in MRM-negative infections can reduce use of the more costly moxifloxacin and potentially lessen the prevalence of antimicrobial resistance. Improved cure rates will likely mean fewer clinic visits for consultations related to additional courses of antimicrobials, potentially reduce the likelihood of developing upper urogenital tract complications, limit spread of infections, and further reduce the need for retesting to document cure.

Quantifying potential cost savings of RGT to justify and guide national policies is a complex matter with very limited data available. Investigators from Australia focused on *M genitalium* infections in cohorts of women, men who have sex with women, and MSM, each with a population of 100,000. They developed dynamic transmission models that compared costs and quality-adjusted life-years (QALYs) gained between RGT and no RGT over a 10-year period to devise a cost-effectiveness ratio on a national level [134]. They utilized epidemiologic data regarding *M genitalium* prevalence, cure rates with first- and second-line treatments, and spontaneous clearance rates and quantified a variety of direct medical costs that included diagnostic testing, resistance testing, clinic visits, numbers of infections treated, antimicrobials, and complications. RGT lowered health care costs with an incremental net monetary benefit of A$1.3 million and higher QALYs in women. The largest cost component was associated with reducing complications of *M genitalium* infection in women, which included PID and chronic pelvic pain. RGT cost savings were significantly higher in MSM with a net monetary benefit of A$17.9 million and higher QALYs. However, in men who have sex with women, RGT was not cost-effective.

This investigation is the first of its kind examining the role of RGT from a health care economist point of view. Its characteristics and findings are unique to Australia and cannot be generalized to other countries where funding sources, cost bases, policies for health care, prevalences of infections and antimicrobial resistance, availability, and prescribing practices of antimicrobials may differ. Only countries with widespread utilization and adherence to RGT and adequate data collection will be suitable for attempting to quantify the value of RGT.

## CONCLUSIONS AND FUTURE DIRECTIONS

A great deal of progress has been made in recent years regarding understanding of *M genitalium* epidemiology, disease associations, laboratory diagnosis, prevalence of antimicrobial resistance, and management. This progress hinges on availability of accurate diagnostic tests. Unfortunately, the United States still lags behind Europe and Australia in terms of the ability to perform RGT and in the availability of repurposed antimicrobial agents suitable as third-line alternatives for MDR *M genitalium* infections. More than six years have passed since FDA clearance of the first NAAT to detect *M genitalium* and 5 years since the CDC recommended inclusion of MRM testing for *M genitalium* as part of RGT in its 2021 STI guidelines, yet there are still no FDA-cleared *M genitalium* NAATs that include MRM testing. Thus, there is still a need for existing CE-IVD–marked resistance tests or other newly developed products to undergo the necessary procedures and clinical trials to gain FDA clearance. Without the availability of commercial kits to detect resistance mutations, none of the following can be performed to the extent that they should be: (1) CDC recommendations for RGT for individualized management of *M genitalium* infections, (2) surveillance for antimicrobial resistance, and (3) studies aimed at assessment of the clinical and economic benefits for RGT.

There is an urgent need to develop inexpensive, rapid (<30 minutes) POC tests that will detect *M genitalium* as well as mutations conferring antimicrobial resistance. To our knowledge, there are currently no products capable of doing this available anywhere in the world, although some products are in development. This would be extremely important in locations with limited resources where complex and expensive laboratory testing is not possible. This need for POC resistance testing was recognized by the US National Institutes of Health in 2024 when a request for proposals was issued to the diagnostic testing industry for partnering with academic institutions to develop such products. Having a POC test would dramatically change the way that RGT is conducted and improve its efficiency. Patients would receive the diagnosis of *M genitalium* infection and the presence or absence of resistance mutations at their initial presentation, eliminating the need for callbacks for treatment orders. POC test availability would make randomized controlled treatment trials feasible to evaluate new antimicrobials and to address such issues as whether combined antimicrobial treatments differ in efficacy from sequential treatments.

Funding for longitudinal national and global surveillance studies and systematic evaluations of patient outcomes with various management strategies should be considered public health priorities, as prevalence, resistance patterns, and responses to antimicrobial chemotherapy can evolve over time, dependent to a large extent on antimicrobial prescribing practices and sexual behaviors. Timely data are also needed to guide periodic updated recommendations regarding *M genitalium* diagnosis and management; to facilitate genotyping studies aimed at improving understanding how antimicrobial is spread within and between populations; and to improve understanding the role of *M genitalium* in certain clinical conditions in specific populations, such as pregnant women and their offspring and women who develop complicated infections of the upper urogenital tract. Management of pregnant women who acquire macrolide-resistant *M genitalium* infections is a dilemma since tetracyclines and fluoroquinolones are not recommended in pregnant women. Current CDC STI guidelines do not address *M genitalium* treatment in pregnant women.

It is important for research and reference laboratories to continue to attempt to culture to obtain fresh clinical isolates of *M genitalium* for genomic and pathogenicity studies and for evaluation of new diagnostic tests and antimicrobial agents. More work is needed to evaluate the clinical significance of the 23S rRNA mutation A2062G and whether it should be included in diagnostic tests. Studies are needed to determine the prevalence and significance of *parC* and *gyrA* mutations in fluoroquinolone treatment failures in the United States to ascertain whether testing for QRMs in addition to MRMs is clinically necessary.

Existing antimicrobials are insufficient to treat all *M genitalium* infections in an adequate manner. This situation is especially dire in the United States, where third-line drugs used in other countries are not available. Obtaining clinical efficacy data for gepotidacin and zoliflodacin in the treatment of *M genitalium* is desirable to determine whether either might be a suitable treatment alternative. Further exploration of existing agents, such as fusidic acid and nitroimidazoles, is also warranted, as is the search for new drugs with unique targets that will not be subject to cross-resistance with existing drug classes. New mechanisms of antimicrobial resistance in *M genitalium* are now being identified, and more are likely to be found in the future if research in this area continues.
